# One Step Double Augmentation with Human Dermis Allograft and Homologous PRP in Misdiagnosed and or Chronic Achilles Tendon Ruptures

**DOI:** 10.1111/os.13871

**Published:** 2023-09-28

**Authors:** Marcello Lughi, Elena Bondioli, Cinzia Moretti, Nicolò Maitan, Matteo Ferretti, Roberto Casadei

**Affiliations:** ^1^ AUSL of the Romagna Ravenna Italy; ^2^ Rizzoli Institute Bologna Italy; ^3^ Malatesta Novello Hospital Cesena Italy

**Keywords:** Achilles tendon, decellularized dermis allograft, Double augmentation, Homologous platelet‐rich plasma (H‐PRP), Misdiagnosed chronic rupture, Regenerative medicine

## Abstract

**Objective:**

Misdiagnosed/chronic Achilles tendon injuries are rare and disabling for patients. The surgical treatment of these rare injuries aims to ensure the tendon heals mechanically and biologically. This is the prerequisite for a good clinical and functional outcome and reduces recurrences. The main aim of the study is to present a surgical technique that has proven to be original, reproducible, and capable of guaranteeing solid tendon repair and optimal tissue regeneration.

**Methods:**

We treated five patients, four males and one female, with the one‐step double augmentation technique. All patients of this study complained of pain, but above all severe functional limitation that Achilles tendon injury had been causing for more than a month. In this study, we widely described the surgical technique, original and not found in the literature, which provides a biological graft (allograft of decellularized dermis) and homologous, thrombin‐activated, platelet‐rich plasma (H‐PRP) in a single step. Surgical approach, always used by the first author, respected predefined steps: careful dissection and preparation of the peritendinous tissues from suture to the end of the procedure, tenorrhaphy, and augmentation with allopatch to obtain a mechanically effective repair to avoid recurrences, and finally “biological” augmentation with a unit of homologous, thrombin activated, PRP. We offered to all patients a regenerative rehabilitation program post‐operatively.

**Results:**

All patients were evaluated clinically (functional clinical tests and questionnaires) and instrumentally (elastic‐sonography and perfusion MRI). The obtained results have been evaluated at a minimum follow‐up of 18 months and a maximum of 24 months. In all patients pain was resolved, and district function and kinetic chains improved with resumption of daily activities, work, and sports.

**Conclusion:**

The present study confirmed the regenerative potential of decellularized dermis allograft and PRP (homologous and thrombin‐activated). The same approach can also be exploited in cases of severe tendon destructuring and limited “intrinsic” regenerative potential at any age. The proposed one‐step surgical technique of a double augmentation therefore appears useful, safe, reproducible, and applicable in all chronic tendon lesions with low regenerative potential.

## Graphical abstract

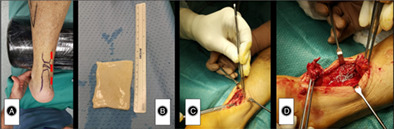

## Introduction

A patient who experiences trauma that occurred at least 4–6 weeks before the orthopedic evaluation, and who reports persistence functional limitation (in daily activities and sport) and pain in the Achilles region has, by definition, a chronic/unrecognized tendon injury. Misdiagnosed/chronic Achilles tendon rupture has an incidence of 10%–25% in all cases of complete tendon injury.^1^ Physical examination is sufficient to confirm tendon rupture with evident structural gap on palpation, a positive Thompson test, with associated bruising and peritendinous soft tissue imbibition.^2^ The patient, of any age, who aspires to an improvement in clinical/functional conditions and in the absence of absolute contraindications, should be treated surgically.^3^ One of the outcomes of surgical treatment is to avoid failure of the repair and clinical relapse.

From the moment of injury to treatment, the tendon tissue and peritenon degenerate and fibrotize.^4^, ^5^ Tenorrhaphy, which must guarantee resistance to tension/traction forces in the immediate post‐operative period and over time, due to local tendon anatomopathological conditions, may not be sufficient. Therefore, it must be implemented in the operative phase using biomaterials to achieve mechanical and biological augmentation. Mechanical augmentation aims to increase the stress resistance of the repair performed, while biological augmentation aims to stimulate the “re‐tendonization” process.^6^, ^7^, ^8^

To date, various types of biomaterials have be used successfully. A distinction is made between synthetic and biological grafts. The second one can be divided into autograft, allograft, and xenograft. The allopatch used is a decellularized collagen membrane derived from human dermis. Light and electron microscopic analysis of this allopatch demonstrates an optimal three‐dimensional collagen structure that is crucial for tissue regeneration. Growth factors such as transforming growth factor beta 1 (TGF beta1) and interleukin‐6 (IL‐6) can also be detected. The structure of the allopatch promotes the anabolic activity of the tenocytes that grow on it. It also has good mechanical properties and histocompatibility.^6^, ^8^, ^9^, ^10^

For a long time, exploiting the regenerative properties of growth factors released *in situ* after application, the use of homologous platelet‐rich plasma (H‐PRP), bone and adipose mesenchymal cells has been envisaged.^11^, ^12^, ^13^

For some time, exploiting the regenerative properties of growth factors released *in situ* after application, the use of homologous platelet‐rich plasma (H‐PRP), bone and adipose mesenchymal cells, has been envisaged in many therapeutic protocols applied to humans.^11^, ^12^, ^13^

A one‐stage and double augmentation surgical approach is not described in the literature. We therefore hypothesized a useful synergy between the mechanical properties of allopatch and the biological regenerative properties of H‐PRP.

Optimized reparative surgery as described above is essential for early optimal loading leading to better clinical and functional outcomes.^14^

The primary aim of the work was to describe the one step double augmentation technique, used to treat misdiagnosed/chronic Achilles tendon injuries.

The technique will have scientific significance if it: (i) demonstrates simplicity of execution, reproducibility, and ductility in different, more or less complex, tendon pathological conditions; and (ii) clinical and functional results remain stable over time with no recurrence.

## Methods

All patients provided their written consent (attached in the medical record) to perform the surgery according to the one stage double augmentation technique. The ethics committee of the AUSL of Romagna with determination No. 2929 in 2021 authorized the evaluation of the results.

The surgical procedure involves performing tenorrhaphy with Bunnell technique reinforced using an allograft, in the form of a patch of decellularized dermis, and stimulating the regeneration process by infiltrating the construct with PRP and thrombin homologues. The intra‐ and post‐operative phase have been planned, focusing on some aspects:

1. Precise pre‐operative and intra‐operative quantization of pathological tendon anatomy.
2. Evaluation of mechanical and biological characteristics of available and usable augmentation systems and surgical technique.
3. Based on the quality of the repair obtained, the post‐operative program was customized trying to apply the modern concepts of optimal loading and neuro‐mio‐functional recovery of the kinetic chains.
4. A minimum of 1.5 years follow‐up evaluation of clinical/functional and anatomical results are obtained and processed.

***Pre‐operative pathological anatomy quantization***

All patients underwent an ultrasound study, a nuclear magnetic resonance imaging and digital x‐ray in anterior–posterior and lateral‐lateral projection in load.

***Intra‐operative pathological anatomy quantization***

Three lesions of the Achilles midportion and one pre‐insertion lesion with fibers disruption in the axial plane were treated. One lesion involved the tendon midportion with fibers rupture in the coronal plane, configuring a tendon slippage.

***Mechanical/biological characteristics of the augments used***

We performed mechanical (and biological) augmentation using as biomaterial a decellularized dermis allopatch. Allopatch processing, and distribution were performed according to national rules on tissues for transplantation. Then, a sample of human dermis was taken from multi‐organ and/or multi‐tissue donors and then transported to the “Regione Emilia Romagna” Skin Bank of Bufalini Hospital, Cesena, Italy for processing; here the tissues were submitted to dermis separation from the epidermidis, decellularization, and storage in nitrogen vapors at −180°C. Twenty‐four hours before surgical implantation, a sample of tissue of requested sizes was prepared, and then sent in a sterile condition from the Skin Bank of Bufalini Hospital to our hospital, and here conserved at a temperature of 4°C until its use. Allopatches can be requested in various sizes (width and length in cm) and thickness (<1 mm–>2 mm).

It was considered that an increase in the regenerative potential could be reached by exploiting the growth factors released by thrombin‐activated PRP, both homologous.^12^ The numerous and well‐known growth factors released upon PRP injection are added to those already present in a dermal allopatch (PGF Beta).^15^

The surgical technique performed involves the following steps: from tenorrhaphy to double one‐step augmentation.

Patient in prone decubitus and pneumo‐ischemic tourniquet at the root of the limb, the tendon lesion is approached with a medial para‐tendon incision. After careful dissection of the subcutaneous layers, the peritenonio is isolated. After removal of the fibrous scar tissue and organized hematoma at the level of the focus of chronic tendon injury, “quantization” of the same was performed by assessing the retraction of the stumps, adherence to the surrounding tissues and the degree of fibrillar degeneration. In complete transverse lesions on axial plane, an end‐to‐end tenorrhaphy by the Bunnell technique was performed. In all cases the tendon was sutured without leaving a gap between the stumps, seeking, because it was deemed useful, an “optimal shortening” repair of the tendon.

To provide greater resistance to tension/traction stresses of the tenorrhaphy, considering the severe tendon destructuring, an onlay augmentation was performed. The repaired tendon was then wrapped with a decellularized dermis allopatch. The suture of the allopatch to the tendon was performed with absorbable #3.0 thread with great attention was made to make it as tight as possible to the tendon. Afterwards a suture to previously isolated peritenon is made, connecting it to the allopatch to optimize vascular support to the construct.

**Fig. 1:**
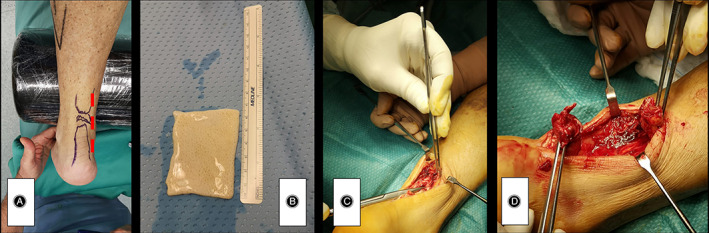
(A) Skin markers of the lesion site and hatched the incision site. (B) Allopatch of dermis. (C) Dissection and retrieval of the peritenon. D) Quantization of the lesion (adhesions, fibrosis, etc.).

**Fig. 2:**
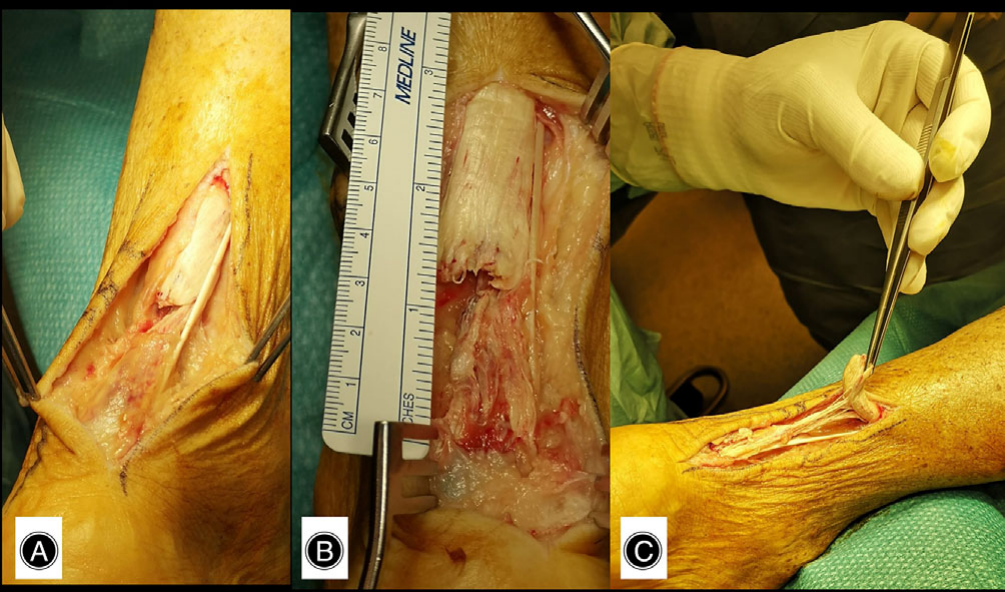
(A–C) Appearance of the tendon slippage‐type lesion.

**Fig. 3:**
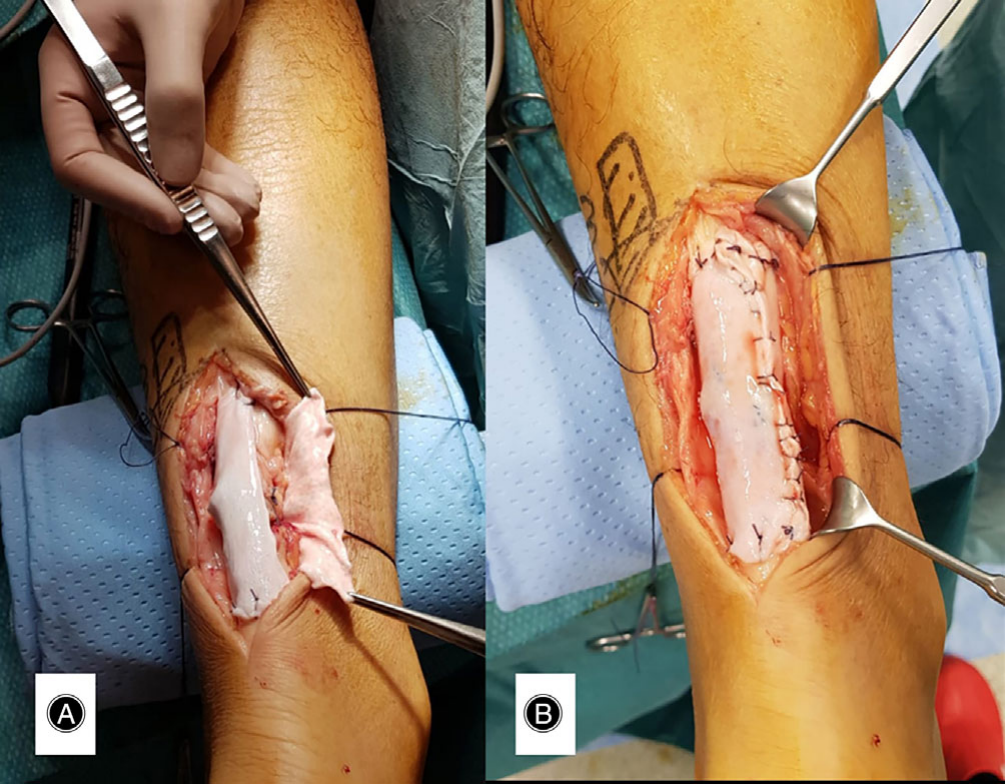
(A) Onlay passage of dermis allopatch. (B) Fully sutured allopatch.

**Fig. 4:**
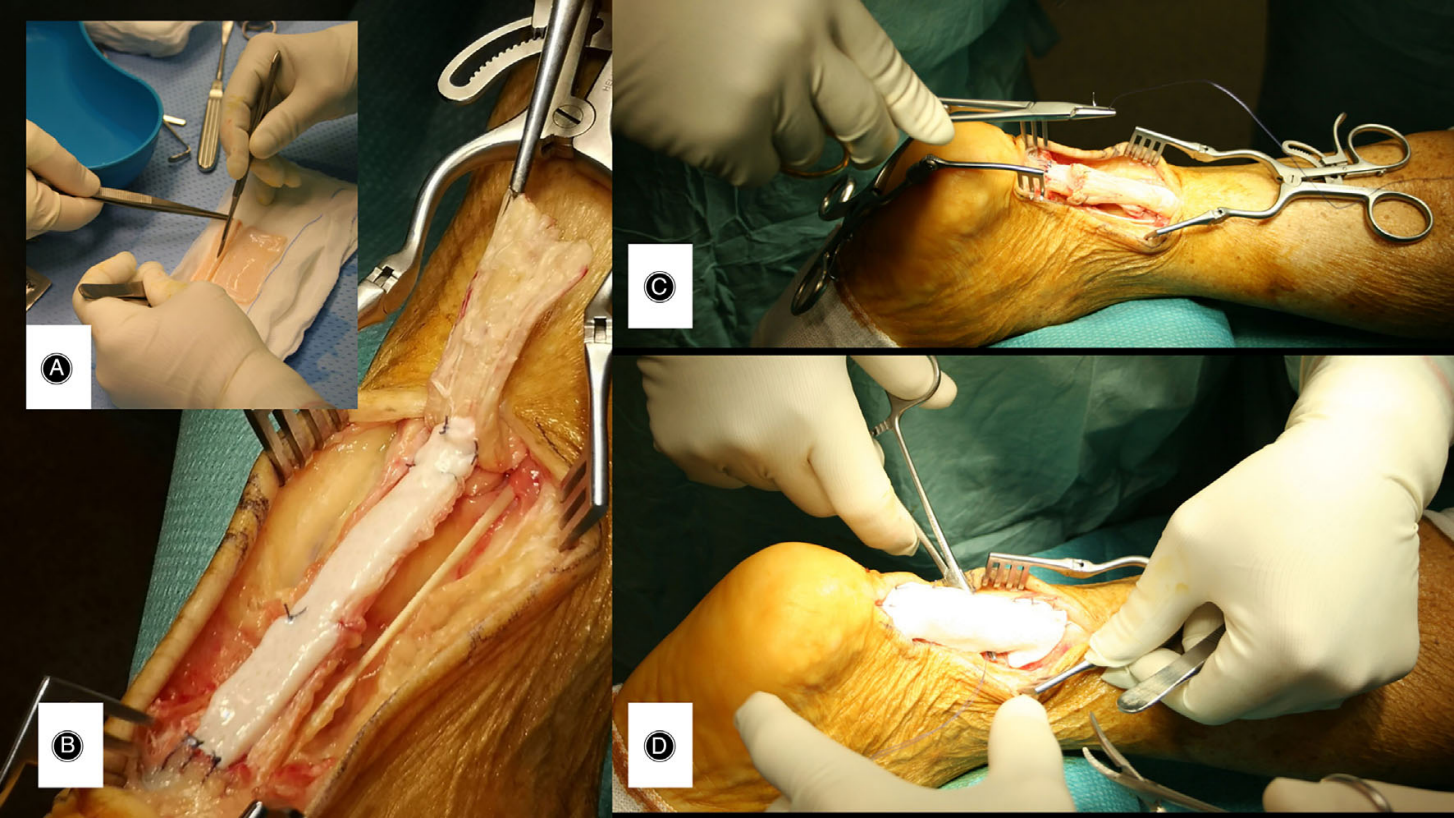
(A–D) Inlay‐Onlay augmentation in tendon slippage injury.

**Fig. 5:**
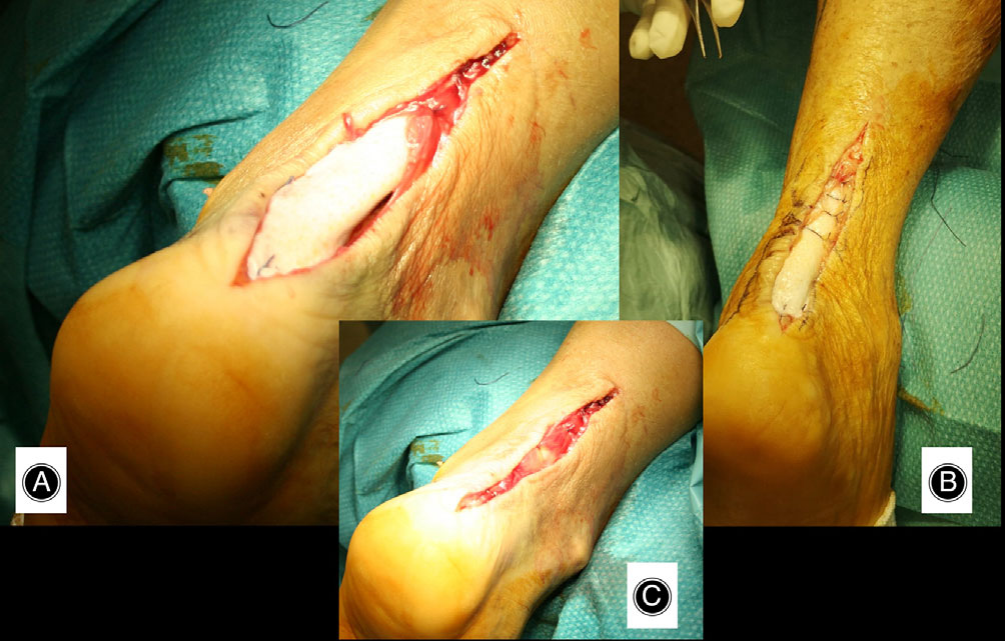
(A–C) Suture of the peritenon.

**Fig. 6:**
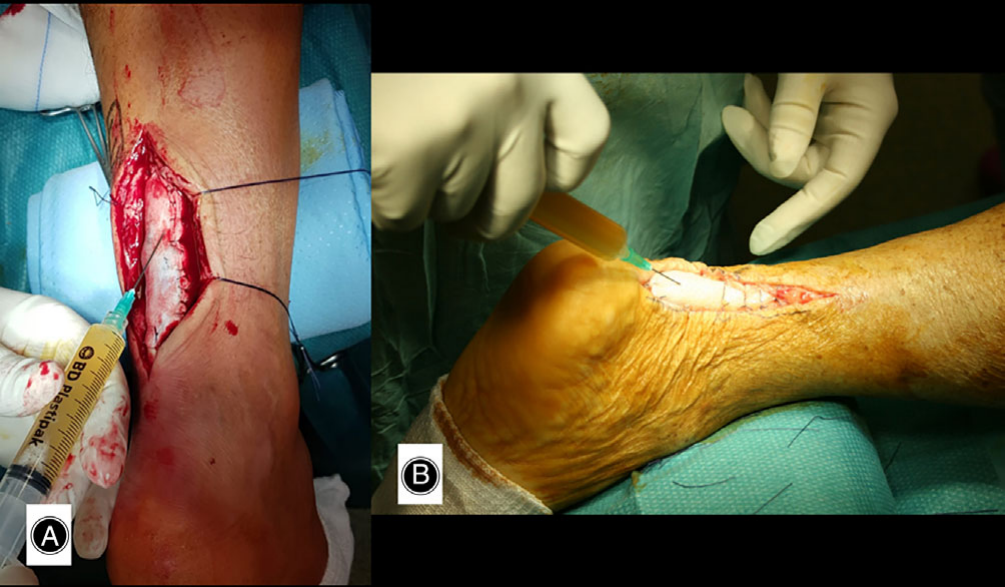
(A, B) Homologous activated PRP infiltration.

At the end of tenorrhaphy/onlay augmentation, infiltration of the “tendon repair biological chamber” was performed, exploiting one unit (6 cc) of PRP activated with 2 cc of thrombin, both homologous. In the lesion with tendon slippage at the midportion, tenorrhaphy and simultaneous augmentation was performed, interposing an allopatch “tape” (same width as the Achilles tendon) between the tendon stumps. In addition to the inlay reparative technique (wafer like repair), an onlay augmentation was performed. As described above, the peritenon was sutured and infiltrated with an activated homologous PRP unit (Figures 1, 2, 3, 4, 5, 6).

In all the studied cases, suture of the skin was performed without excessive tension because the deformation of the anatomical profile of the tendon at the end of the procedure was not such as to represent a skin covering problem. After applying a hydro fiber dressing, a boot with a series of wedges inside (three wedges of approximately 10° each one) was used to ensure equinization of approximately 30°.

### Post‐operative Protocol

In all treated patients a construct considered solid and able to be solicited according to the most modern rehabilitation concepts, such as regenerative rehabilitation was obtained.^14^ The post‐operative rehabilitation program must to combine “respect” of the tendon healing process and optimal loads, which have proven to be “regenerative” for the tendon. Generally, the re‐educational steps follow one after the other, according to the objectives that have been anticipated and achieved, considering that joint and muscle work must not trigger pain. Final objectives include the recovery of range of motion (especially of ankle and knee), strength of all the elements of kinetic chain, normal motor, and functional patterns, and at the end, return to activity, to play and to sport.

All patients have been banned from loading for 10 days. After that, patients are allowed to walk with full weight bearing and pain tolerance. Every 7 days, a wedge is removed from the walker. Once all three wedges have been removed, patients begin a dry and in‐water functional re‐education program. For 15 days after performing exercises, patients reapply the walker. On the 45th day, patients completely remove walker and continue the functional re‐education program of the district and kinetic chains.

Postoperative instrumental evaluation at least 8 months after surgery was conducted employing the elastosonographic technique and dynamic perfusion MRI study.

The ultrasound‐elastosonographic study was conducted using Hitachi‐Aloka Arietta V70 ultrasound, 5–18 MHz linear probe, and Real Time elastographic module with strain mode.

**Fig. 7:**
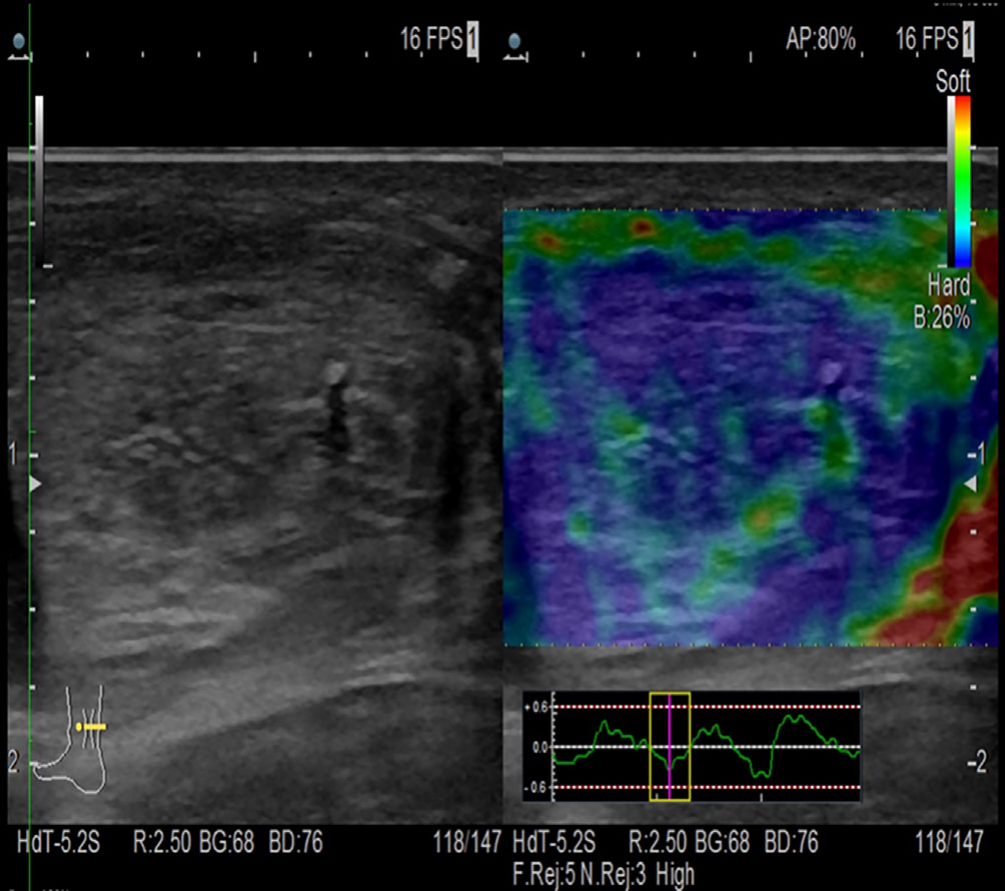
In the left part of the image: B‐Mode ultrasound study. In the dx part of the image: elastosonographic study with good representation of blue areas demonstrating recovered tendon stiffness.

The elastosonographic technique integrates the classic B‐Mode ultrasound study, which is useful for the evaluation of the fibrillar structure of the tendon, with data inherent biomechanical properties (stiffness and elasticity) of the tissues. The elastosogram with gradations of blue to indicate tendon stiffness of the repaired tissue similar to physiological stiffness and red to indicate suboptimal stiffness and thus greater tendency for elongation with consequent risk of tendon damage (tissue not yet fully “regenerated”)^16^ (Figure 7).

The MRI study was conducted on Philips Achieva dStream tomograph operating at 1.5 T with mDixon TSE T2 acquisitions in the sagittal plane (TE 80 ms, TR 3600 ms, Echo Train Length 23, Number of Signal Averages 1), TSE T1 (TE 20, TR 560, ETL 7, NSA 1) and TSE T2 (TE 80, TR 3100, ETL 38, NSA 1) in the axial plane, and THRIVE in the axial plane (TE 2, TR 3, ETL 30, Flip Angle 10, NSA 1) for dynamic contrastographic evaluation, administering by means of a double head injector (Nemoto Sonic Shot GX) a paramagnetic contrast medium bolus (gadoterate, 0.1 mmol/kg) followed by a thrust bolus of saline (20 ml), Injected at the rate of 2 ml/s through agocannula into a right cubital vein. Surface receiving antenna was used and scans were set in comparison with the healthy—untreated side.

**Fig. 8:**
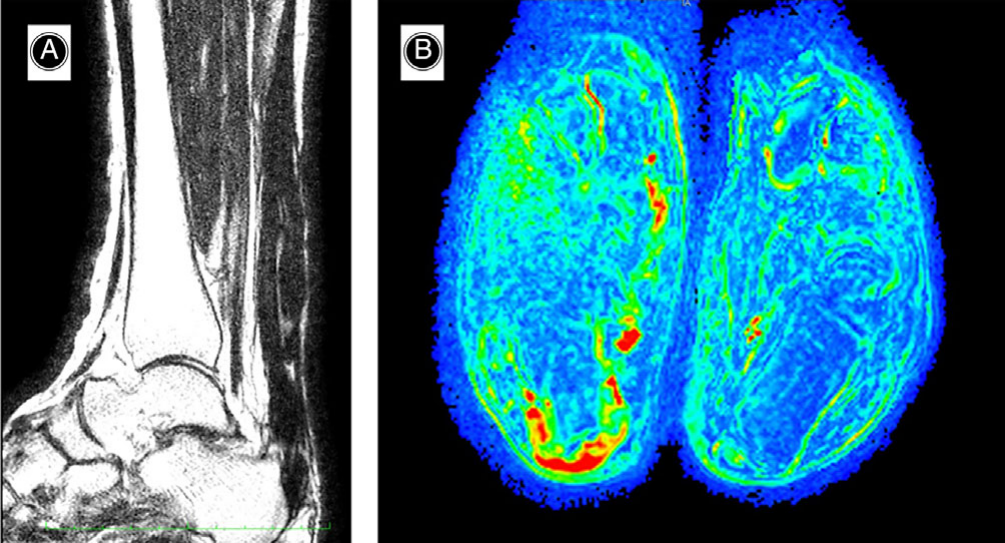
(A) Conventional sagittal MRI study with altered Achilles tendon signal. (B) Dynamic perfusion MRI study. Signs of incomplete tendon healing are evident in the left image. Right image healthy side.

The dynamic perfusion MRI study was performed by T1 scanning repeated several times over time after intravenous injection of paramagnetic contrast agent (gadolinium), which progressively accumulates in the extracellular space with a rate determined by the phenomena of perfusion and capillary permeability and correlated with vascular surface area. The dynamic MRI study integrates the conventional study with a quantitative analysis of contrast medium uptake after processing through specific models. These analyses are very useful already at the first scheduled checkup by comparing the treated tendon with healthy tissue. They are also useful especially in subsequent checks at time to evaluate the evolution of “regenerative” processes. Dynamic contrastographic MRI study allows an assessment of enhancement that testifies to the extent and degree of granulation tissue maturation related to the reparative and graft integration phenomena.^17^ (Figure 8).

A good correlation between elastosonographic and MRI data was observed in defining regions of decreased stiffness and increased perfusion indices that were considered representative of a still evolving process of allopatch integration and maturation and “tendon regeneration.”

## Results

In the period between January and December 2020, five patients (four males and one female) have been treated for chronic/misdiagnosed Achilles tendon injuries. Their age ranged from 35 to 78 years. Patients were evaluated with a follow‐up at 18–24 months. Three patients were still in work and engaged in leisure‐time sports activities. All patients had not recovered functionally and clinically after trauma, during sport or in the daily activity, that occurred at least four‐six weeks prior to orthopedic evaluation. Table 1 shows Achille's tendon lesions location.

**TABLE 1:** Achille's tendon lesions location and types

*Patient*	Tendon lesion location	Type of lesion (plane)
#1	Achilles midportion	Axial plane
#2	Achilles midportion	Axial plane
#3	Achilles midportion	Axial plane
#4	Achilles midportion	Coronal plane
#5	Pre‐insertional portion	Axial plane

Reduction in function was accompanied by pain in the hindfoot and edema of the calf. In view of the marked deterioration in Quality of Life (QoL) reported by all patients and in absence of absolute contraindications, no age limits on surgical indication were placed.

The small number of patients and their different post‐surgical rehabilitation compliance led us to prepare a “personalized” evaluation sheet that highlighted the most significant aspects from an anatomical, clinical, functional and subjective point of view (Table 2).

**TABLE 2:** Outcomes of clinical and functional parameters

Clinical and functional parameters	#1	#2	#3	#4	#5
Continuity of the Achilles tendon (ultrasound and MRI)	Yes	Yes	Yes	Yes	Yes
Correct tension of the Achilles tendon Thompson test negative	Neg	Neg	Neg	Neg	Neg
Correct tension of the Achilles tendon Matles test negative	Neg	Neg	Neg	Neg	Neg
Pain (numeric rating Score 0–10)	0	0	0	1–2	1–2
Neurological and Skin complication	No	No	No	No	No
Active Ankle ROM recovery (sagittal plane) Operated vs. health side (symmetric or asymmetric)	Yes	Yes	Yes	No	No
Passive Ankle ROM recovery (sagittal plane) Operated vs. health side (symmetric or asymmetric)	Yes	Yes	Yes	No	No
Daily activity recovery Walking without pain Walking without lameness Climbing and descending stairs	Yes	Yes	Yes	Yes	Yes
Sport activity at the same level	Yes	Yes	Yes	No	No
Patient satisfaction	Yes	Yes	Yes	Yes	Yes
Strength recovery calf muscle Standing calf rise test on 2 legs (10 reps)	10	10	10	4	3
Strength recovery calf muscle Standing calf rise test on 1 legs (10 reps)	10	10	10	2	0
Strength recovery calf muscle Standing calf rise test endurance (range 15–25 reps)	Yes	Yes	Yes	No	No
Neuro‐myo‐functional full recovery	Yes	Yes	Yes	No	No

The “ultrastructural” evaluation with standard MRI + perfusion and elasto‐sonography was performed at least 8 months after surgery, especially for those younger patients who had joined the proposed rehabilitation program. In these patients, MRI demonstrated optimal integration, regeneration and remodeling of repaired tissue.

**Fig. 9:**
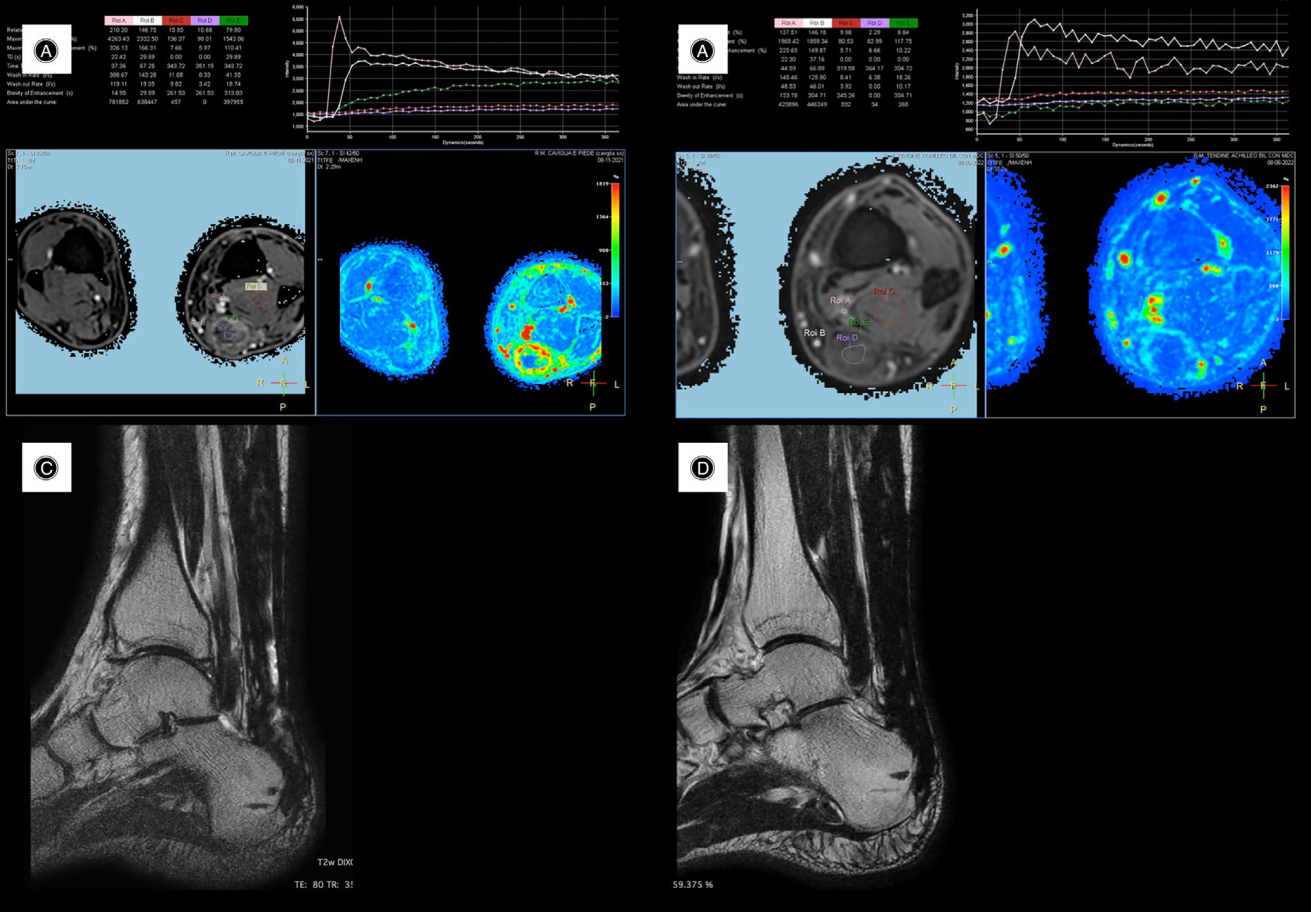
(A) Dynamic‐perfusion MRI at 12 months after surgical treatment. Incomplete and evolving tendon “regeneration.” Green‐red signal. (B) Dynamic‐perfusion MRI at 18 months after surgical treatment. Complete tendon “regeneration.” Blue signal. (C) Conventional MRI in sagittal T1 at 12 months after surgical treatment. Uneven signal of the tendon however continuous. (D) Conventional sagittal T1 MRI at 18 months after surgical treatment. Good restoration of tendon structure.

The best instrumental results were obtained in midportion lesions rather than pre‐insertional lesions. In older and patients not very adherent to the rehabilitation program, the MRI and elastosonographic study showed good ultrastructural recovery at 18 months (Figure 9).

Recovery of calf strength and function were tested with the heel‐rise test performed on one and on both limbs. Younger patients performed the test correctly and did not demonstrate exhaustibility of strength. Older patients, on the other hand, were shown to “compensate” for the heel‐rise test deficit of the operated limb by exploiting the force of the contralateral. All patients recovered function by climbing and descending stairs and walking without pain, lameness, and distance limitations. In all patients, the Thompson and Matles test was negative and there were no gaps on palpation of the Achilles tendon. Apart from one (elderly) patient, all the others recovered sports activity at the same level of pre‐trauma performance. All patients reported to be satisfied with the obtained results reached with the surgery they underwent.

## Discussion

Treatment of a chronic/misdiagnosed Achilles tendon injury represents a real challenge for orthopedic surgeon who has to operate tissues of reduced quality, widely degenerated, with poor intrinsic potential to heal, even after surgery, and possibility of failure of the performed suture.^11^, ^18^, ^19^, ^20^ It is a rare disease, which can affect patients of different ages, functional requirements, and compliances.

Surgical solution is mandatory if one wants to restore function, recovery of daily living and sporting activities and also reducing pain.^3^ “Regeneration” of repaired tendon is the basis to achieve the aforementioned objectives. In these anatomical‐pathological conditions (chronic degeneration), application of mechanically solid tenorrhaphy that confer resistance to tension stress is not sufficient. Especially in the cases which involve severe tissue destructuring, so tendon suture augmentation procedures should be considered.^6^, ^7^, ^8^

From the analysis of literature augmentation methods we can exploit tissue adjacent to the tendon to be repaired (V‐Y advancement technique, tendon transfers) or involve the use of grafts divided into autografts (fascia Lata, hamstring tendons), allografts (membranes of various sizes and thicknesses) and synthetic material (Gore‐Tex, polyurethan urea, polylactic acid, etc.).^21^, ^22^

Analyzing the advantages and disadvantage of the above‐mentioned augmentation methods the first one may be associated with morbidity problems at the harvest site. Allografts and synthetic products are preferable because they do not pose problems at the sampling site, they are readily available even if it has to be considered that cost problems may arise.^6^, ^7^, ^9^, ^10^ Allografts are the most widely used biomaterials, with many clinical applications including soft tissue reconstruction and sports medicine.^23^, ^24^ Their mechanical and biological/regenerative aspects have been extensively analyzed.^6^, ^7^, ^8^, ^9^, ^20^, ^25^, ^26^, ^27^, ^28^ An ideal allopatch should be biocompatible, it must not interfere with nominal phases of the healing process (inflammation, regeneration and remodeling), it should be easily integrated into the surrounding tissues, it must be mechanically resistant and act as a true scaffold re‐inhabited by vessels (neo‐generated and/or from paratenon), by cells (neo‐generated and/or from peritenon) and by collagen tissue, and it should be manageable in its use in intra‐operative phase.

Allogeneic transplants (allografts) can be divided into:

1. Unprocessed allograft;
2. Decellularized allograft; and
3. Xenograft (e.g. porcine small intestinal submucosa, bovine pericardium).^24^

Our study is a proposal for mechanical/biological double augmentation. The allograft used is a hydrated membrane of decellularized matrix of human dermis. The decellularization process can be performed in various methods.^15^, ^18^, ^23^, ^29^ The physical–chemical decellularization process used by our dermis bank involves the use of trypsin and low‐dose gamma radiation. Structural, microscopic, and biological evaluations have shown that allopatch maintains intact the extracellular structure (collagen fibers, glucosaminoglycans and proteins) which acts as a temporary mechanical support; at the same time, it stimulates cellular and vascular growth (TGF beta 1 remains active). The patches are three‐dimensional structure membranes that demonstrate *in vitro* and *in vivo* integration, biocompatibility and predisposition to remodeling during healing process, which confers high load‐bearing resistance.^15^, ^30^ It is authors’ opinion that it might be useful to exploit the regenerative biological potential of H‐PRP. Although conclusions of many RCTs have not provided univocal indications, the role of growth factors derived from the degranulation of concentrated platelets in the healing process of the Achilles tendon which is well known. IL‐6, TGF beta, BMP, etc. have shown promising results in tissue regeneration.^4^, ^11^ They increase concentration of growth factors in the allopatch level increasing potential of cell adhesion, proliferation (fibroblasts) and differentiation.^20^

For many years homologous thrombin activated PRP has been used, produced from donors' blood, in the treatment of osteoarthritis (especially of knee and ankle) and in acute soft tissue injuries (skin and tendons) with any healing problems. Whole blood units intended for PRP/Thrombin production are selected based on platelet count (>200 per microliter), AB positive blood group. Bags from donors taking drugs that may adversely affect platelet function are discarded. The concentration procedure with closed‐loop systems guarantees sterility and prevent any infectious diseases. Centrifugation reaches minimum values of 1 million concentrated platelets (as established by Italian legislation) and few leukocytes. Finally, units produced are frozen.^11^, ^12^, ^13^, ^31^ It is considered by the authors that the double augmentation method used met the desired characteristics and aims required by the applied regenerative medicine. One‐step application of allopatch and PRP, also based on evaluation of the literature, is original. The post‐operative rehabilitation program is crucial and influences the healing process (as a “third” augment). Early mobilization and administration of right loads promote healing and recovery of strength.^14^ The Achilles tendon is a structure that is “re‐educable and regenerates,” if precise rehabilitation programs are followed.^5^ Evaluation of the regenerative “re‐tendinization” of the repaired area must be conducted with instrumental investigations (elastosonography and MRI with contrast medium) that highlight any healing with pure fibrous tissue.^16^, ^17^

## Conclusion

Although the small number and heterogeneity of operated patients does not allow any consideration with statistical significance, it can be said that age plays a fundamental role in post‐operative recovery. The young patients are certainly more compliant than the elderly and the global ultrastructural tendon structure and the neuro‐myo‐functional “reserve” are precarious in the latter.

Adherence to the rehabilitation program is also more correct in the younger patient who has more motivation and greater functional demands. The optimal post‐operative loading, avoiding functional mechanical overloads, better integrates, reorganizes, and reshapes the allopatch.

Although the perfusional MRI study and elasto‐sonography allow the evaluation of post‐surgical “structural remodeling and reorganization” with good clinical correlation, histological studies in second look surgery would be desirable even it often not applicable due to obstructive ethical rules.

Given the good results obtained, the intent of the authors is to also apply the proposed surgical technique in acute lesions, especially in patients who, with pre‐operative MRI, demonstrate to have a severely and extensively degenerated Achilles tendon.

In conclusion even if it was possible to conduct a purely descriptive study, it can be stated that:

- use of the dermis patch provides a valid mechanical and biological support;
- dermis patch is biocompatible and integrable to the Achilles tendon;
- the role of homologous PRP cannot be judged but its role as a booster of biological healing cannot be excluded;
- rehabilitation program seems to guide the structural evolution of the tendon. Well re‐educated and rehabilitated patients had good clinical and instrumental findings; and
- topography of the lesion appears to be correlated to an optimal clinical and instrumental evolution. Midportion lesions have a good chance of recovering clinically and structurally.

Based on these considerations, tenorrhaphy plus one step double augmentation is considered a reproducible, safe, and effective method to treat misdiagnosed and chronic Achilles tendon lesions.

## Author Contributions

Marcello Lughi is the surgeon who conceived and performed all the operations. He conceived and drafted the manuscript. Cinzia Moretti is the doctor in charge of the transfusion center at the Forlì hospital of the AUSL Romagna that provides us with the H‐PRP units. Matteo Ferretti is the radiologist who performed the instrumental examinations (elastosonography and MRI). Elena Bondioli is the doctor in charge of the Derma Bank at the Cesena Hospital of the AUSL Romagna. Nicolò Maitan contributed to the drafting of the manuscript and bibliographic research. Roberto Casadei is the doctor in charge of the Traumatology Operative Unit of the Forlì Hospital of the AUSL Romagna and contributed to the drafting of the manuscript and collaborated in the execution of the surgical procedures.

## Ethics Statement

Ethics approval and consent to participate. The study was authorized by the ethics committee of the AUSL Romagna (CEROM). Determination n° 2929. Study participants provided the first author, the surgeon performing all procedures on all participating patients, with written informed consent to participate in the study. The derma allopatch used was produced in accordance with current regulations.

## Data Availability

The Datasets generated during and/or analyzed during the current study are not publicly available due privacy policies but are available from the first author on reasonable request.
